# Electrocardiogram Biometrics Using Transformer’s Self-Attention Mechanism for Sequence Pair Feature Extractor and Flexible Enrollment Scope Identification

**DOI:** 10.3390/s22093446

**Published:** 2022-04-30

**Authors:** Kai Jye Chee, Dzati Athiar Ramli

**Affiliations:** School of Electrical and Electronic Engineering, USM Engineering Campus, Universiti Sains Malaysia, Nibong Tebal 14300, Malaysia; kai_jye@student.usm.my

**Keywords:** transformer, BERT, ECG biometrics, self-attention mechanism, deep learning, multi-class classification, convolutional neural network, feature extraction, blind segmentation, artificial neural network

## Abstract

The existing electrocardiogram (ECG) biometrics do not perform well when ECG changes after the enrollment phase because the feature extraction is not able to relate ECG collected during enrollment and ECG collected during classification. In this research, we propose the sequence pair feature extractor, inspired by Bidirectional Encoder Representations from Transformers (BERT)’s sentence pair task, to obtain a dynamic representation of a pair of ECGs. We also propose using the self-attention mechanism of the transformer to draw an inter-identity relationship when performing ECG identification tasks. The model was trained once with datasets built from 10 ECG databases, and then, it was applied to six other ECG databases without retraining. We emphasize the significance of the time separation between enrollment and classification when presenting the results. The model scored 96.20%, 100.0%, 99.91%, 96.09%, 96.35%, and 98.10% identification accuracy on MIT-BIH Atrial Fibrillation Database (AFDB), Combined measurement of ECG, Breathing and Seismocardiograms (CEBSDB), MIT-BIH Normal Sinus Rhythm Database (NSRDB), MIT-BIH ST Change Database (STDB), ECG-ID Database (ECGIDDB), and PTB Diagnostic ECG Database (PTBDB), respectively, over a short time separation. The model scored 92.70% and 64.16% identification accuracy on ECGIDDB and PTBDB, respectively, over a long time separation, which is a significant improvement compared to state-of-the-art methods.

## 1. Introduction

Identification and verification are very important concepts in surveillance and security systems [1]. Conventional approaches, whether they are knowledge-based, or token-based, are susceptible to loss and transfer [2,3,4]. Biometrics-based methods aim to sidestep these problems by using the intrinsic characteristics of the human body, such as the fingerprint, iris, voice, face, keystroke, and gait [5,6]. Despite having their own strengths and weaknesses [7,8], some of them have made it to real-world applications [3]. The electrocardiogram (ECG) has enough interperson variability (intervariability) to be used as biometrics [9]. As a bonus, liveness information is inherent to the ECG signal [3,4].

### 1.1. Electrocardiogram

The ECG is a representation of the electrical activities of the heart [10]. Electrical signals generated by the polarization and depolarization of the cardiac tissue can be detected by electrodes, called leads, attached to the skin surface of various body parts [8,11]. Plotting the data against time reveals the ECG.

The obvious features in the ECG are the P wave, the QRS complex, and the T wave. The P wave is formed from the combination of the depolarizations of the right atrium and then the left atrium, while the QRS complex corresponds to the depolarizations of the right ventricles and then the left ventricles, and the T wave represents the ventricular repolarizations [11]. The time interval between two consecutive R peaks is called the R-R interval [12].

In a typical ECG processing application, a raw ECG signal is transformed into representations suitable for the classifier to work on. This process is called feature extraction, and it is performed either by conventional feature extraction algorithms or by human expert knowledge [13]. As deep learning gains popularity, the feature extraction task is sometimes taken over by artificial neural networks.

### 1.2. Identification and Verification

Since both the verification and the identification are classification problems, in this paper, the term “classification” is used to refer to both at the same time.

Before any classification, the system needs to be informed with a set of identities to be considered for the classification. This is done through enrollment which refers to the process of registering a new identity into the system [14]. In terms of ECG biometrics, a new identity enrolls by giving up a sample of its ECG. A digitized ECG signal is denoted as G, and the data point sequence that constitutes G is denoted as $\left(g_{1},g_{2},\ldots ,g_{n}\right)$, where n is the total number of data points. Depending on the system’s design, the G may be processed [15] before it is stored [14] for classification later. The enrolled identities become the scope for consideration during the classification phase. Ordinal numbers are used as labels for the identities in a scope. Therefore, a scope is represented as S={1,2,…,h}, while the ECGs in the scope are represented as $J=\left\{G_{1},G_{2},\ldots ,G_{h}\right\}$, where h is the total number of people.

An unknown identity that needs to be verified or identified is called a query [14,15,16,17], and it is denoted as q with its ECG denoted as $G_{q}$. In the process of individual verification, first, an enrolled identity, k, is claimed [3], then, the system verifies if the claim is true [14], typically by calculating a score or probability using the equation below:

(1) $$P\left(q=k\right)=f_{VE}\left(G_{q},G_{k}\right)$$

where $f_{VE}$ is an arbitrary verification function and k∈S.

Individual verification can be generalized into scope verification [15,16]. In this case, if q matches one of the identities in S, then it is considered. This probability is calculated by the equation below:

(2) $$P\left(q\in S\right)=f_{VE}\left(G_{q},J\right)$$

In closed identification, q must be in S, so the identification can be expressed as a probability mass function:

(3) $$P_{q}\left(k\right)=f_{ID}\left(k,G_{q},J\right)$$

where $f_{ID}$ is a closed identification function, k=1,2,…,h and $\sum_{k=1}^{h}P_{q}\left(k\right)=1$.

For practical applications, open identification is needed [15,16,17], where P(q∉S)>0. This task can be achieved by combining the results of the closed identification and the scope identification. The related terminologies and their descriptions are summarized in Table 1.

## 2. Related Works

This section first presents the evaluation metrics used in the ECG biometrics literature before presenting the other related research works.

### 2.1. Evaluation Metrics

Metrics are used to evaluate the performance of an ECG biometric system. For some of the metrics, different terms are used among researchers to refer to the same metrics. Table 2 shows the metrics used in this research, alternative terms used by other researchers, and the metrics’ descriptions.

### 2.2. Related Works on ECG Biometric

Sellami et al. [11] use public databases, namely MITDB, NSRDB, ECGIDDB, and STAFFIII, for their research. Raw ECG signals are transformed using Discrete wavelet transform (DWT), and the features are selected and stored in the system. To verify a person, template matching is used to find the correlation between stored features and query features. To identify a person, template matching is performed between the query and every enrolled person; the highest score is considered the identified person.

Ingale et al. [14] investigate and compare the performance of verification systems built with different filters, segmentation methods, feature extraction methods, and classification methods. For filters, the Kalman filter and infinite impulse response (IIR) filter are tested. For segmentation, they test on R peak to R peak (R-R) and fixed window around an R peak. For fiducial features, 30 are selected, while Symmlet and Daubechies wavelet transformation are used for non-fiducial features. For classification, they test Euclidean distance and dynamic time warping (DTW). All the designs are tested with five public databases and one private database. The results of the different combinations of methods are reported. A total of 10 ECG segments are required for enrollment. Authentication lengths vary with different databases, but the lengths are not documented in the paper.

Pal et al. [19] use Finite Impulse Response (FIR) equiripple filters to remove baseline wander noise, power interference noise, and high-frequency noise. They use Haar wavelet transform to delineate the ECG signal before extracting fiducial features, which they categorize into interval features, amplitude features, angle features, and area features. Then, they use principal components analysis (PCA) and kernel principal components analysis (KPCA) for dimensionality reduction and calculate Euclidean distance for matching.

Tan et al. [5] filter by first transforming the ECG signals with fast Fourier transform (FFT), applying the bandpass filter, and then Inverse FFT to obtain the filtered signals. They use a moving window to find local maxima to detect R-peak. To improve the feature extraction accuracy, they remove some of the outliers. From here, two sets of feature extraction methods and classification methods are used in sequence. The first one extracts a total of 51 fiducial features and then uses the random forest classifier. The second one decomposes the ECG using DWT and 1-to-S template matching based on wavelet coefficients, where S is the reduced number of candidates based on the probabilities calculated from the random forest classifier.

In the research by Li et al. [21], the ECG is segmented by detecting R-peak and taking a fixed-length around the peak. They train a convolutional neural network which they call F-convolutional neural network (F-CNN) to extract ECG features. The F-CNN is trained using the FANTASIA database, where its goal is to identify 1 of the 40 people given one heartbeat. The last two layers of the F-CNN are discarded, and the vector produced is considered the ECG features. M-convolutional neural network (M-CNN), the second part of their model, uses the features from two heartbeats (one from the query person and the other from the enrolled person) to compute a matching score. The enrollment requires 100 heartbeats to generate a template for each person. Without retraining, the cascaded CNN can work with CEBSDB, NSRDB, STDB, and AFDB.

In research by Sun et al. [6], they specifically mention the time separation between the enrollment and classification. PTBDB and ECGIDDB are used because they have, on average, 63 days and 9 days of time separations between multiple recording sessions, respectively. They filter the ECG using the Butterworth filter and IIR filter. The blind segmentation method is used. They make sure the segments are gathered from different recording sessions that have obvious time separation. Multiple domain analysis methods are used to extract the ECG features. The mean, standard deviation, kurtosis, and skewness represent the features in the time domain. Mel-frequency cepstral coefficients (MFCCs), FFT, and Discrete cosine transform (DCT) are the features from the frequency domain. As for the features in the energy domain, they use discrete Teager energy operators. They introduce the channel attention module (CAM) into the convolutional neural network to be used as their classifier. They use 40 s for enrollment and 4 s for identification.

Salloum et al. [22] use ECGIDDB and MITDB for their research. Fixed-width segmentation around the R peaks is used to obtain heartbeats. They design their model using the RNN. The enrollment and classification both require 18 heartbeats, and each heartbeat is treated as a time step in a sequence.

Labati et al. [18] propose to use CNN for ECG biometric recognition, named Deep-ECG. They filter the signal using an IIR filter and then segment by taking 0.125 s around the R peak. R peaks are located using an automatic labeling tool. They train a CNN for feature extraction and identification. Deep-ECG can also verify a person by computing the distance between two heartbeat templates.

Zhang et al. [23] propose the HeartID. They filtered the raw ECG data with the Butterworth bandpass filter and then scaled the data into a range of 0 to 1. They used 2 s blind segmentation and then used autocorrelation to remove phase shift from the blind segmentation. They used DWT for feature extraction and 1D-CNN for classification. CEBSDB, WECG, FANTASIA, NSRDB, STDB, MITDB, AFDB, and VFDB were used for training and testing.

All the reviewed related works are summarized in Table 3.

## 3. Problem Statement

Four problems are explored further in this research: independent feature extraction, inability to capture inter-identity relationships, fixed enrollment scope, and insufficient training data.

### 3.1. Independent Feature Extraction

ECG changes even in the same person. The ECG amplitude and heart rate can change due to mental, emotional, physical, and health conditions [23,24] and measuring conditions such as the placement of electrodes and devices [8,24,25]. These changes affect some of the fiducial features [8,11]. More importantly, ECG can be different depending on the time of measurement [2,6,24,26]. This means that the accuracy decreases as the time separation between the enrollment and the classification increases. However, this problem is not addressed properly. For instance, Li et al. [21] experiment with a very short time separation between enrollment and classification, while Tan and Perkowski [5] randomly choose heartbeats for enrollment and classification.

Sun et al. [6] show that there are time-related features in the ECG, and feature extraction based on these features can improve the model accuracy. However, the feature extraction methods we have seen so far work independently in the enrollment phase and classification phase. Given an enrolled ECG as $G_{k}$ and a query’s ECG as $G_{q}$, the extracted features for these two ECGs are computed as in (4) and (5), respectively.

(4) $$L_{k}=f_{FE}\left(G_{k}\right)$$

(5) $$L_{q}=f_{FE}\left(G_{q}\right)$$

where $L_{k}$ is the enrolled feature vector, $L_{q}$ is the query feature vector and $f_{FE}$ is the feature extraction function. Any time-related features between $G_{k}$ and $G_{q}$ are impossible to extract by independent feature extraction.

### 3.2. Inability to Capture Inter-Identity Relationship

Identification is a multi-class classification problem; every enrolled identity is a class. One approach is to reduce an identification to multiple verifications between the query and every enrolled identity and then compare the verification probability at the end. Every probability for the event of q matching an identity is expressed as:

(6) $$\left\{P_{q}\left(1\right),P_{q}\left(2\right),\ldots ,P_{q}\left(h\right)\right\}=f_{P}\left(p_{1},p_{2},\ldots ,p_{h}\right)$$

where each p is a verification probability against a person in the identification scope and $f_{P}$ is a function that normalizes all the inputs into a probability distribution like SoftMax. This approach is flexible to scope changes because enrolling or removing identities does not require retraining the model. However, due to each verification only having conditions on the corresponding enrolled ECG and the query ECG, it is unaware of the whole identification scope (scope agnostic). This is a significant drawback due to the inability to capture the relationship between different classes [27,28,29]. There are researchers trying to turn SVM, a binary classifier by design, into a multi-class classifier [30,31], and others are trying to improve the reduction approach by injecting extra information [32,33]. Luo [34] even suggests that introducing new subclasses in some cases can improve a multi-class classifier.

### 3.3. Fixed Enrollment Scope

Another approach to the identification task is to use a compatible multi-class classifier to compute the probability distributions over all classes internally. A classifier is trained on a fixed enrollment scope. The ability to identify with that scope is intrinsic to the model, thus making it scope-aware. However, this means that the design is inflexible to scope changes as retraining is required to accommodate new identities.

Li et al. [21] and Labati et al. [18] design and train their multi-class models and then modify them into binary models just for the benefit of flexibility. There is a dilemma of choosing between accuracy or flexibility.

### 3.4. Insufficient Training Data

Many of the publicly available ECG databases either have a low number of people in the database, each with longer recordings, or have more people, each with shorter recordings. As a result, attempting to split a single database into training, testing, and, optionally, validation datasets is challenging. Some models seem to do well with larger training sets, but that leaves only a small set of data for testing. For instance, the most accurate model by Salloum et al. [22] uses up to 80% of the data for training. Moreover, if the ECG is segmented by heartbeat, the data are further limited by the number of heartbeats in the recording.

Combining multiple databases to increase the dataset is difficult because it needs to reconcile the differences across databases, potentially having to deal with different measuring devices, measuring conditions, sampling rate, type of noise, etc. This could be the reason why training a single model using multiple databases is unpopular. However, if this could be done, it would not only increase the training dataset size but could also generalize the model by capturing a wider range of ECG variations.

## 4. Novelty Contributions

We propose a novel ECG pair feature extractor, $f_{EP}$, to replace the independent feature extraction described in Section 3.1. Joint feature vectors of the query and the enrolled, $L_{kq}$, are extracted $f_{EP}$ by conditioning on both $G_{k}$ and $G_{q}$ in a single process. Since $G_{k}$ and $G_{q}$ are separated by time, $L_{kq}$ contains time-related features of the ECG pair. Equation (7) summarizes the process of the ECG pair feature extractor.

(7) $$L_{kq}=f_{EP}\left(G_{k},G_{q}\right)$$

The ECG pair feature extractor is inspired by the sentence pair feature extraction of BERT. However, we do not employ the pre-training and fine-tuning technique. Instead, two different feature vectors are produced by the ECG pair feature extractor, $L_{kq\left(\mathrm{VE}\right)}$ is used for the identification task and $L_{kq\left(\mathrm{ID}\right)}$ is used for the verification task:

(8) $$L_{kq}=\left\{L_{kq\left(\mathrm{VE}\right)},L_{kq\left(\mathrm{ID}\right)}\right\}$$

We propose a novel identification encoder (ID encoder) to be used as the classifier for the identification. It uses the encoder in the transformer to function as a true multi-class classifier because the self-attention mechanism captures the inter-identity relationship. This solves the problem described in Section 3.2. Since the transformer is designed for variable-size input, the ID encoder can accept any classification scope as input, so it is flexible to scope changes without retraining, which solves the problem in Section 3.3.

We propose a novel dataset generation procedure by using blind segmentation as a data augmentation technique. This procedure is not limited by the number of heartbeats in the ECG recording. We also propose combining multiple ECG databases to increase the total number of people and to provide more ECG variations. A total of 10 databases were used to generate the training and validation dataset, and another six databases were used to evaluate the model. The huge amount of data with wide variations trained a generalized model and solved the problem described in Section 3.4.

## 5. Materials and Methods

This section first explains the details of the data pre-processing and the dataset generation procedure. Then, it explains the details of the model design. Finally, the training specs and metrics are documented.

### 5.1. Databases

The 10 ECG databases in Table 4 are publicly available on Physionet [35] and were chosen for the model training. These databases contain ECG recordings from healthy people, as well as people with heart conditions.

### 5.2. Pre-Processing

Pre-processing is important in reshaping the ECG signals into a specific format that the model expects. The pre-processing used are resampling, segmentation, filtering, and standardizing. Resampling and segmentation are required for datasets generation because most databases have different sampling rates and recording lengths. In a real-world application, if an ECG is recorded at the correct sampling rate and length, resampling and segmentation can be omitted, but filtering is recommended, and standardizing is always required.

- Resampling. We choose to train the model to operate on 128 Hz ECG data because this frequency is relatively low even for most wearable devices [21].
- Segmentation. Blind segmentation is used [6,23], so no fiducial points are needed. Moreover, blind segmentation directly reflects the data collection time, which is an important specification to consider for a practical application. The segment length is 3 s because 3 s per classification is still practical in a real application. Each segment has 384 data points after being multiplied with a 128 Hz sampling rate.
- Filtering. We employ a fifth-order Butterworth bandpass filter to denoise the ECG segments. $0.01f_{N}$ and $0.7f_{N}$ are the lower and upper critical frequencies of the bandpass filter where $f_{N}=64\;\mathrm{Hz}$. It is important to segment the signal before filtering because filtering creates distortions at both ends of the signals, which must not be ignored in an actual classification scenario.
- Standardizing. We employ the standard score normalization, referred to as standardizing, to every ECG segment, G, including all the ECG segments in the scope and the query ECG segment. Each point in the segment, g, is transformed to $g^{\prime }$ by:

(9) $$g^{\prime }=\frac{g-\mu }{\sigma }$$

where μ and σ are the mean and standard deviation of G, respectively.

### 5.3. Training and Validation Datasets Generation Procedure

First, the identities in the databases are split into a training group and a validation group according to the training–validation split ratio column specified in Table 4. Then, the ECG recordings are resampled to 128 Hz. After that, the single example generation (Algorithm 1) is repeated 2,580,480 times on the training group to obtain 2,580,480 training examples. Likewise, Algorithm 1 is repeated 32,768 times on the validation group to obtain 32,768 validation examples.

The single example generator (Algorithm 1) is the proposed novel dataset generation procedure. An example consists of J and $G_{q}$ as the input and the true identity of q as the label. In step 1, a database is randomly chosen, then, 32 identities are randomly chosen from that database, and they are assigned as S. This step ensures that every database has an equal chance of appearing in the dataset. If the chosen database has less than 32 identities, step 2 through step 6 fill up the remaining identities from other random databases. Step 7 randomly selects an identity from S and assigns it as q. Step 8 through step 14 contain the ECG segmentation. These steps ensure that $G_{k}$ and $G_{q}$ are not overlapping. Step 15 filters all the ECG segments. Step 16 standardizes all the ECG segments.
**Algorithm 1.** Single example generator.1S ← 32 random identities from 1 random database2**while** size of S is less than 32:3   db ← random database4   k ← random identity from db5   **if** k is not in S:6       add identity to S
7q← random identity from S8J ← empty set9for each k in S:10   if k is equal q:11      $G_{k}$$,\;G_{q}$ ← 2 random ECG segments without overlapped12   **else**:13      $G_{k}$ ← random ECG segment14   $\;\mathrm{add}\;G_{k}\;\mathrm{to}\;J$
15$\mathrm{filter}\;J\;\mathrm{and}\;G_{q}$16$\mathrm{standardize}\;J\;\mathrm{and}\;G_{q}$17**return**$\;J,\;G_{q},\;q$

### 5.4. The Model

**Figure 1:**
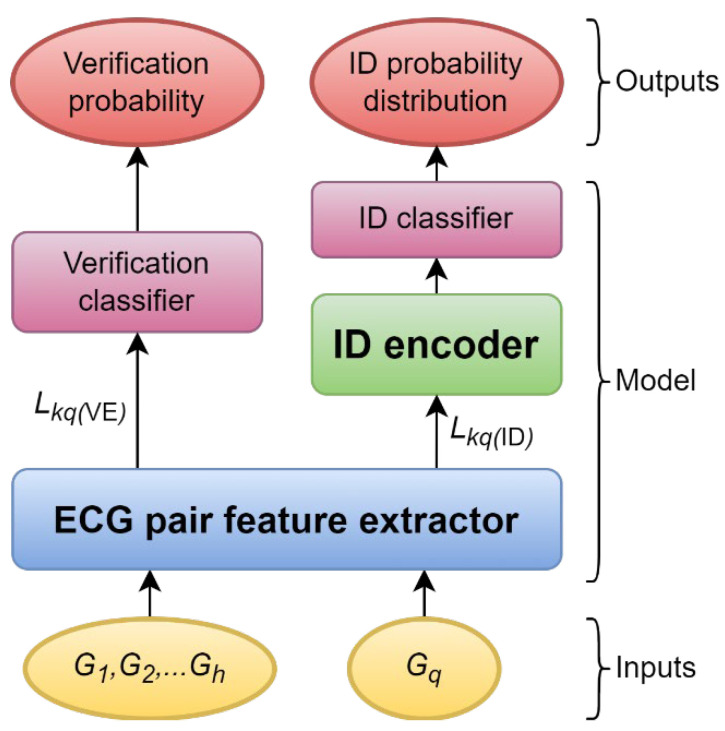
An overview of the model design.

The inputs of the model are the classification scope ECGs, J, and the query ECG, $G_{q}$. The ECG pair feature extractor extracts features of J and $G_{q}$, the details are explained in Section 5.4.1. Using the extracted features, the model performs verification and identification at the same time. The features are processed by the verification classifier, which is explained in Section 5.4.5, and the outputs are the probabilities of q matches each of the enrolled identities. As for the identification, the features are processed by the ID encoder, which is explained in Section 5.4.6 and the ID classifier, which is explained in Section 5.4.7, and the output is a probability distribution for all the enrolled identities. Figure 1 shows that the model consists of an ECG pair feature extractor, verification classifier, ID encoder, and ID classifier.

#### 5.4.1. ECG Pair Feature Extractor

**Figure 2:**
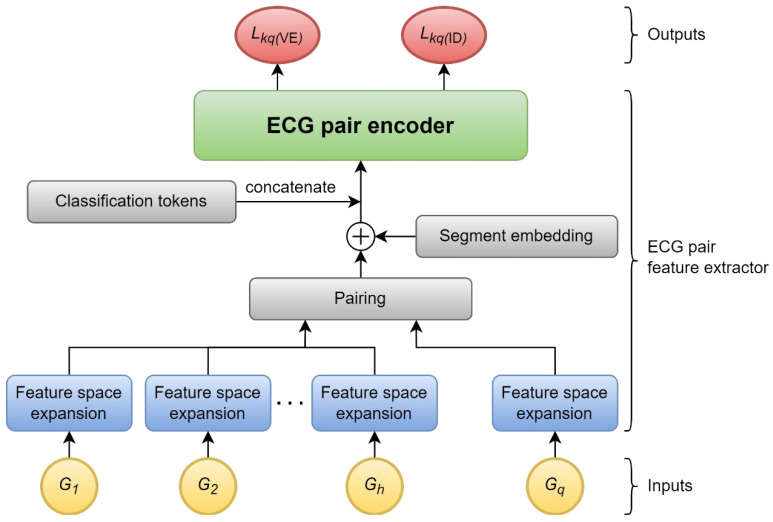
ECG pair feature extractor.

The key idea in the ECG pair feature extractor is to use BERT’s sequence pair encoder to find information in an ECG pair. Figure 2 shows the components of the ECG pair feature extractor and how the ECGs are processed to become the feature vectors. Every ECG is processed by the feature space expansion into a sequence, and the details are explained in Section 5.4.2. Then, the query sequence is paired with each enrolled sequence, added to the segment embedding information, and concatenated with classification tokens. These 3 processes are explained in Section 5.4.3. Finally, the ECG pair encoder, explained in Section 5.4.4, performs self-attention on the sequence to produce 2 feature vectors.

#### 5.4.2. Feature Space Expansion

The feature space expansion replaces the sub-word embedding in the original transformer to reshape an ECG into a sequence. The feature space expansion consists of a 1D convolutional layer with Rectified Linear Unit (ReLU) activation and a 1D max-pooling layer. The convolutional layer has 512 filters with a kernel size of 33 and operates at a stride of 1. The max-pooling layer has a kernel size of 16 and operates at a stride of 16. An input $G\in {\mathbb{R}}^{384}$ is expanded into $X\in {\mathbb{R}}^{22\times 512}$. All the enrolled ECGs and the query ECG are expanded by the same process resulting in $X_{1},X_{2},\ldots ,X_{h}$ and $X_{q}$.

#### 5.4.3. Pairing, Segment Embedding, and Classification Tokens

**Figure 3:**
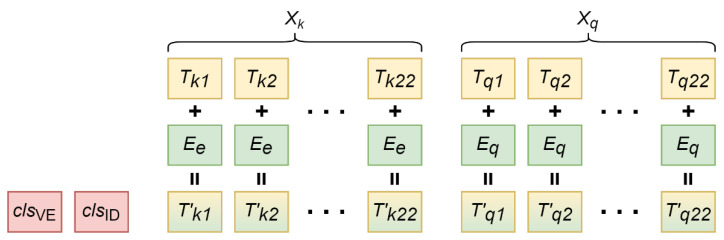
A composite sequence consists of 2 classification tokens, an enrolled sequence, and the query sequence. $E_{e}$ is added to each element in $X_{k}=\left(T_{k1},T_{k2},\ldots ,T_{k22}\right)$ resulting in $\left({T^{\prime }}_{k1},{T^{\prime }}_{k2},\ldots ,{T^{\prime }}_{k22}\right)$. $E_{q}$ is added to each element in $X_{q}=\left(T_{q1},T_{q2},\ldots ,T_{q22}\right)$ resulting in $\left({T^{\prime }}_{q1},{T^{\prime }}_{q2},\ldots ,{T^{\prime }}_{q22}\right)$.

$X_{q}$ is duplicated h times so that it can be evenly paired up with $X_{k}$ where k=1,2,…,h. A trainable enrolled segment embedding vector, $E_{e}$, is added to every element in $X_{k}$. A trainable query segment embedding vector, $E_{q}$, is added to every element in $X_{q}$. Two trainable classification tokens, $cls_{\mathrm{VE}}\in {\mathbb{R}}^{512}$ and $cls_{\mathrm{ID}}\in {\mathbb{R}}^{512}$, are prepended to the sequence. At this point, we have h composite sequences; each sequence is $X_{kq}\in {\mathbb{R}}^{46\times 512}$. Figure 3 illustrates the process of pairing the expanded ECGs and injecting the sequence with segment embeddings.

#### 5.4.4. ECG Pair Encoder

**Figure 4:**
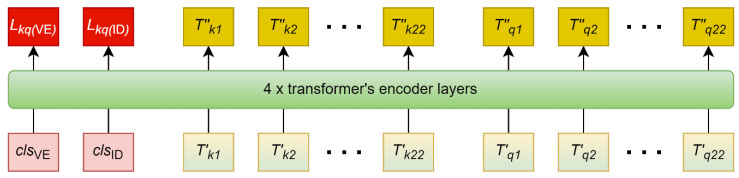
The ECG pair encoder is adapted from Bidirectional Encoder Representations from Transformers (BERT)’s sequence pair encoder. It extracts joint features from the two input ECG sequences. ${T^{\prime \prime }}_{k1},{T^{\prime \prime }}_{k2},\ldots ,{T^{\prime \prime }}_{k22}$ are the final hidden states that correspond to ${T^{\prime }}_{k1},{T^{\prime }}_{k2},\ldots ,{T^{\prime }}_{k22}$ respectively; ${T^{\prime \prime }}_{q1},{T^{\prime \prime }}_{q2},\ldots ,T{T^{\prime \prime }}_{q22}$ are the final hidden states that correspond to ${T^{\prime }}_{q1},{T^{\prime }}_{q2},\ldots ,{T^{\prime }}_{q22}$ respectively. $L_{kq\left(\mathrm{VE}\right)}$ is the final hidden state that correspond to $cls_{\mathrm{VE}}$; $L_{kq\left(\mathrm{ID}\right)}$ is the final hidden state that correspond to $cls_{\mathrm{ID}}$.

The ECG pair encoder consists of 4 transformers’ encoder layers. $d_{model}=512$ is used, which is the same as the base model transformer in [36]. Figure 4 shows that every composite sequence output from the processes in Section 5.4.3 goes through the ECG pair encoder. The final hidden vectors at positions corresponding to $cls_{\mathrm{VE}}$ and $cls_{\mathrm{ID}}$ are the extracted feature vectors, $L_{kq\left(\mathrm{VE}\right)}$ and $L_{kq\left(\mathrm{ID}\right)}$, where k=1, 2,…,h. The self-attention mechanism draws relationships between all tokens in the sequence, causing the feature vectors to have a combined representation of the ECG pair.

#### 5.4.5. Verification Classifier

The input to the verification classifier is $L_{kq\left(\mathrm{VE}\right)}$ from the ECG pair encoder described in Section 5.4.4. The verification classifier consists of four 512-unit fully connected layers, one 256-unit fully connected layer, and one 128-unit fully connected layer. A batch normalization layer and the ReLU activation layer are placed after each of these fully connected layers. A single-unit output layer, a batch normalization layer, and the sigmoid activation layer are used to calculate the verification probability of the query against every identity in the classification scope, P(q=k), where k=1, 2,…,h.

#### 5.4.6. ID Encoder

**Figure 5:**
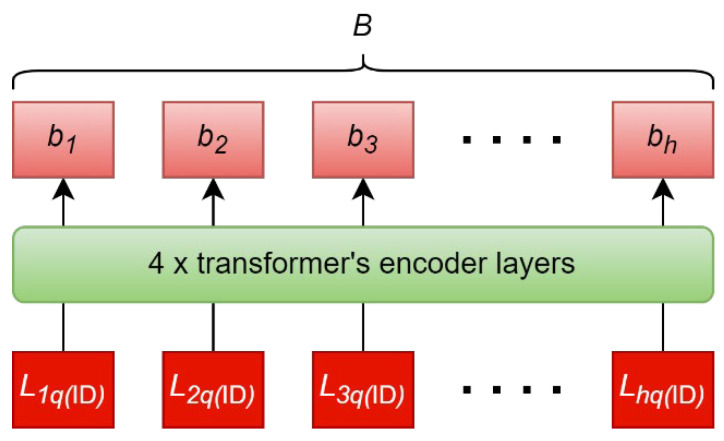
All elements in $L_{kq\left(\mathrm{ID}\right)}=\left(L_{1q\left(\mathrm{ID}\right)},L_{2q\left(\mathrm{ID}\right)},\ldots ,L_{hq\left(\mathrm{ID}\right)}\right)$ form the input sequence to the ID encoder. The self-attention mechanism draws inter-identity relationships to produce the output sequence, $B=\left(b_{1},b_{2},\ldots ,b_{h}\right)$. $b_{1},b_{2},\ldots ,b_{h}$ are the final hidden states that corresponds to enrolled identity 1, enrolled identity 2,…, enrolled identity h.

The ID encoder consists of 4 transformers’ encoder layers, as shown in Figure 5. $d_{model}=512$ is used, which is the same as the base model transformer in [36]. The feature vector, $L_{kq\left(\mathrm{ID}\right)}$, from ECG pair encoder, as described in Section 5.4.4, forms the input sequence, $L_{1q\left(\mathrm{ID}\right)},L_{2q\left(\mathrm{ID}\right)},\ldots ,L_{hq\left(\mathrm{ID}\right)}$ to the ID encoder. This sequence contains the information of the query and all identities in the classification scope for the self-attention mechanism to draw inter-identity relationships. The output sequence is $B=\left(b_{1},b_{2},\ldots ,b_{h}\right)$, which is used by the ID classifier to calculate the identification probability distribution. The ID encoder can process any number of enrolled identities, h, so enrolling new identities or removing existing identities is possible without retraining.

#### 5.4.7. ID Classifier

ID classifier consists of a 256-unit fully connected layer, a batch normalization layer, and the ReLU activation layer, followed by a single-unit output layer and a batch normalization layer. Every element in $B=\left\{b_{1},b_{2},\ldots ,b_{h}\right\}$ goes through the same layers to produce a logit. SoftMax is used to normalize the logits into the identification probability distribution, $P_{q}\left(k\right),\;k=1,\;2,\ldots ,h$, where $\sum_{k=1}^{h}P_{q}\left(k\right)=1$.

### 5.5. Training

**Figure 6:**
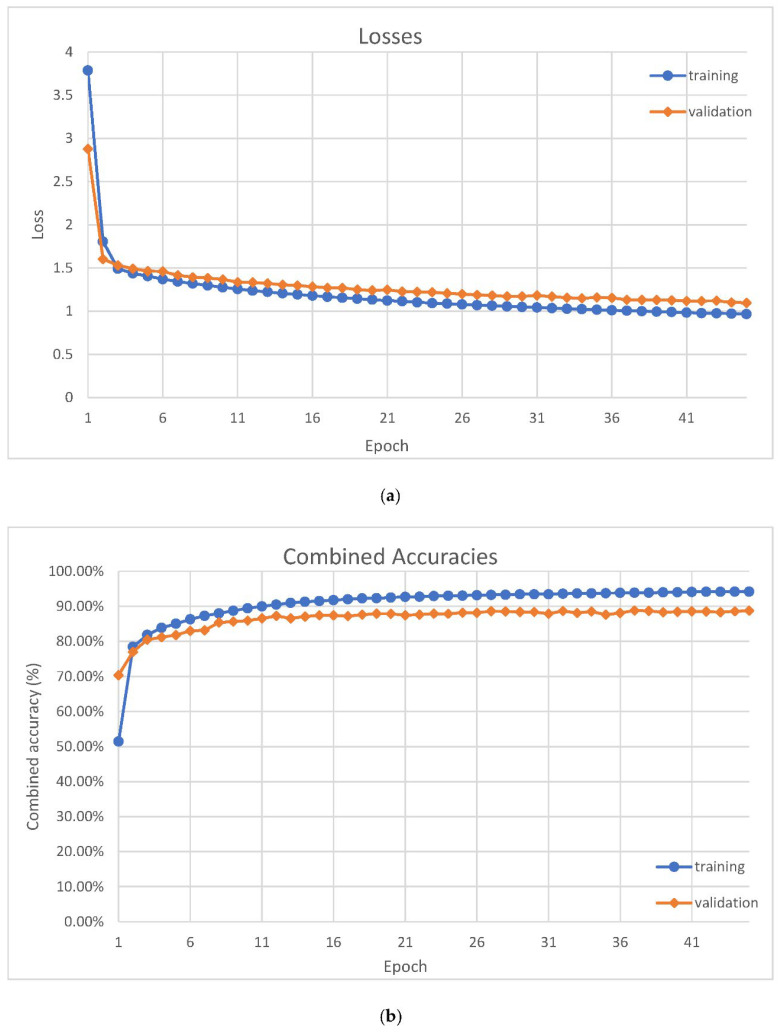
(**a**) Losses are plotted against epoch throughout the training. (**b**) Combined accuracies are plotted against epoch throughout the training. A combined accuracy is the mean of the verification true positive rate (TPR), verification false positive rate (FPR), and the identification accuracy.

We train on the training dataset with 2,580,480 training examples. The dataset is repeated when all training examples are iterated. Each training epoch contains 256 training steps, and each training step uses a batch size of 512. The model’s loss and accuracy are evaluated after each epoch with the validation dataset. The training stops when the validation loss is not improved for 3 consecutive epochs because stopping too early causes undertraining, and training for too many epochs causes overtraining. In our experiment, the training stops at epoch 45. Figure 6a shows the losses, and Figure 6b shows the combined accuracies. A combined accuracy is the mean of the verification TPR, verification FPR, and the identification accuracy.

#### 5.5.1. Optimizer

We use the Adam optimizer [37] with $\beta_{1}=0.9$, $\beta_{2}=0.98$ and $\epsilon =10^{-9}$. We vary the learning rate over the course of training with respect to epoch number, according to Formula (10):

(10) $$0.000012e^{2-0.03epoch}+0.00008$$

#### 5.5.2. Regularization Techniques

During training, we apply dropout to the output of each sublayer of the ECG pair encoder and identification encoder same as the original transformer with $P_{drop}=0.1$. We also smooth [38] all our target labels by $\epsilon_{ls}=0.1$. For the verification task, true label=0.95 and false label=0.05. For identification, true label=0.903125 and false label=0.003125.

### 5.6. Post-Processing

#### 5.6.1. Voting System

Although the model is designed and trained to process 3 s ECG segments, we can fully utilize enrollment ECGs longer than 3 s with a voting system. Enrollment ECGs are split into a v number of 3 s segments, allowing overlaps, to produce v classification results (votes). For closed identification, the most voted identity is considered the final identified. Likewise, the final individual verification also depends on votes. In the case of equal votes, the largest mean probability wins.

#### 5.6.2. Scope Verification

After the final closed identification and individual verification are obtained through the voting system, the scope verification is determined by checking the final individual verification of the final identified position.

### 5.7. Experiment Setup

#### 5.7.1. Enrollment Length, Time Separation, and Classification Window

Time separation between enrollment and classification cannot be ignored when evaluating ECG biometrics because the time separations are real, and they affect the accuracy in practical applications.

**Figure 7:**
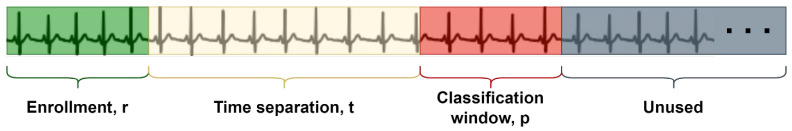
A long ECG recording is divided into enrollment, time separation, and classification window.

For the experiment, a long continuous ECG recording is divided into enrollment and the classification window, as shown in Figure 7. The length of the ECG recording for enrollment is called the enrollment length, r, and it is measured in seconds. The time separation, t, is the time passed from the enrollment phase until the classification phase. The classification window is a portion of the ECG recording where n classification ECG segments are sampled. The length of the classification window is denoted as p, and it is also measured in seconds. This method of dividing the ECG recording allows the same enrollment to be tested at the same t for n times.

#### 5.7.2. Test Databases

A total of 6 databases (Table 5) are selected to test our model. The data have neither appeared in the training dataset nor in the validation dataset. AFDB, NSRDB, and STDB all have a long continuous ECG recording for every person. CEBSDB has 3 recordings recorded in 3 different positions for each person, but they are measured consecutively, so we treat them as long recordings and process them the same way as the other 3 databases. The enrollment, time separation, and classification window are defined, as shown in Figure 7.

For PTBDB and ECGIDDB, only the people with multiple recordings and valid time of measurement are considered in our test. The average time separations are 83.9 days and 5.5 days for PTBDB and ECGIDDB, respectively. Although recordings in PTBDB are at least 32 s, we limit r=32 s. All recordings in ECGIDDB are 20 s, so we use r=20 s.

#### 5.7.3. Short Time Separation Test

Since most of the research in this literature either use very short time separations or completely ignore this variable, this test allows us to fairly compare the results. For AFDB, NSRDB, STDB, and CEBSDB, t=0 is used. For PTBDB and ECGIDDB, the earliest recording is the enrollment, and the second earliest recording is the classification window. The other variables are in Table 6.

#### 5.7.4. Long Time Separation Test

Only PTBDB and ECGIDDB are used for this test. The earliest recording is the enrollment, whereas the latest recording is the classification window. The other variables are in Table 7.

#### 5.7.5. All Time Separations Test

We also test the model by varying t for an insight into its performance against time. Only AFDB, NSRDB, STDB, and CEBSDB are used for this test because they have continuous recordings for each identity. Other variables are in Table 8. The performance of the model is presented as a graph of the metrics in Table 9 against t.

#### 5.7.6. Metrics

When evaluating the model’s individual verification performance, the TPR when FPR is at 1%, 5%, and 10%, the EER, and the area under ROC curve are observed. When evaluating the model’s scope verification performance, the TPR when FPR is at 10%, 20%, and 30%, the EER, and the area under ROC curve are observed. When evaluating the model’s closed identification, the accuracy is observed.

## 6. Results and Discussion

The results from the short time separation test, long time separation test, and all time separation test are described in Section 5.7.3, Section 5.7.4 and Section 5.7.5 are documented and discussed. These results are then compared with the results from other state-of-the-art methods in Section 6.4.

### 6.1. Short Time Separation Test

The model is tested over short time separation, and the results are summarized in Table 10. Not all the results presented have comparable state-of-the-art results, but they could be used in future research comparisons.

The results in Table 10 show that the model performs well in verification and identification even though it is trained once and applied to six databases with different measuring conditions, heart conditions, and number of people.

For individual verification, the model achieves more than 90% TPR at 1% FPR. Practically, this means that it is user-friendly to use at an acceptable FPR. The model also has low EER at less than 4% and a high area under ROC curve at more than 0.9926 in all the databases, which shows its potential to perform under these conditions. The results show that scope verification is more difficult compared to individual verification. However, it is still achieving more than 80% TPR at 10% FPR, less than 16% EER, and more than 0.9226 area under ROC curve across all the databases. The model also achieves higher than 96% identification accuracy across all the databases. We provide the ROC curves for these verification tests in Appendix A to support the results in Table 10, as well as provide all TPR against FPR for future research comparison.

### 6.2. Long Time Separation Test

The model is tested over a long time separation, and the results are summarized in Table 11. Only the identification accuracies have their equivalent state-of-the-art comparison, but individual and scope verification results are documented for future research comparison.

The model performance drops significantly when the time separation between the enrollment and classification increases. However, the model is still able to achieve more than 69% for TPR when FPR is at 1%, and less than 11% EER for individual verification. For scope verification, the model obtains more than 49% TPR at 10% FPR, and less than 28% EER. The model identifies at more than 64% accuracy. We provide the ROC curves for these verification tests in Appendix A to support the results in Table 11, and they also provide all TPR against FPR for future research comparison.

### 6.3. All Time Separation Test

The model performance for all time separation tests is presented as a metric against the time separation graph in Appendix A. This research is the first in the literature to present results in this format, and it could be used for future research comparison. It is important to evaluate a model against all the time separations instead of choosing the best-performing time separation only. Generally, the model’s performance decreases as the time separation increases.

### 6.4. Performance Comparison with Other Methods

In this section, the model performance in this research is compared with the state-of-the-art methods. The results are grouped by test databases for more meaningful comparisons instead of aggregating results from multiple databases as in [21,23].

#### 6.4.1. Individual Verification over Short Time Separation

Table 12 shows the performance of individual verification over short time separation performed using various methods.

Our design underperforms specialized designs of Ingale et al. at CEBSDB, ECGIDDB, and PTBDB. Since Ingale et al. test different combinations of segmentation, filter, and feature extraction, we choose the best results for comparison. The best combination for CEBSDB is fixed-width segmentation, IIR filter, and fiducial features; for ECGIDDB, there is fixed-width segmentation, IIR filter, and DTW features; for PTBDB, there is R-R segmentation, Kalman filter, and fiducial features. It is also worth noting that, for PTBDB, we use different ECG recordings for enrollment and classification, but Ingale et al. use the same recording sessions for both.

Our design underperforms the RNN design of Salloum et al. [22] at ECGIDDB in terms of EER. However, to achieve 0% EER, they use up to 80% of the 89 subjects in the database for training, leaving 20% for testing. Our design outperforms the PCA design of A. Pal and Y. N. Singh at PTBDB.

#### 6.4.2. Closed Identification over Short Time Separation

Table 13 shows the performance comparison of closed identification over short time separation using various methods.

Our design outperforms HeartID of Zhang et al. and cascaded CNN of Li et al. at AFDB, CEBSDB, NSRDB, and STDB. HeartID is a specialized design, i.e., one model is trained for one database. However, the cascaded CNN is a generalized design in that the testing databases are completely separated from the training databases, which is closer to our design.

The random forest design of Tan et al. [5] performs the best at NSRDB and ECGIDDB. However, they randomly select 67% of the extracted heartbeats for enrollment. This means that some of the enrollment lengths could span a long period of time. For instance, some of the recordings in ECGIDDB are 6 months apart, which means that randomly selected heartbeats from these recordings may spread over 6 months.

Our design outperforms the DWT design of Sellami et al. [11] at ECGIDDB, but they only select 40 subjects for testing. Our design slightly underperforms compared with the RNN design of Salloum et al. [22] at ECGIDDB, which also uses different ECG recordings for enrollment and classification.

Our design underperforms compared with the Deep-ECG of R. D. Labati at PTBDB, which they only tested on 52 healthy subjects, and it is not clear if they use the same or different recordings for enrollment and classification.

#### 6.4.3. Closed Identification over Long Time Separation

Table 14 shows a performance comparison of the closed identification over a long time separation with the CNN design of Sun et al.

Our design shows a 6.76% increase in identification accuracy for ECGIDDB and a 7.23% increase for PTBDB. Although the models are trained specific to databases, they are multi-class classification designs like ours. Therefore, the significant performance increase supports the fact that our ECG pair feature extractor can extract time-related features from the query ECG and enrolled ECG and that these features are necessary when the time separation is long.

## 7. Conclusions

In this work, we have adapted the transformer to perform identification and verification using ECG as biometrics. Using BERT’s sequence pair training concept, the ECG pair feature extractor can extract dynamic features from an ECG pair. Using the transformer’s encoder as a multi-class classifier, this design analyzes the entire identification scope, and at the same time, it is also flexible to the scope changes without retraining.

We have also proposed a dataset generation method based on blind segmentation that is not restricted by the number of heartbeats in a recording. Using this method on 10 publicly available ECG databases, a huge training dataset is generated. This satisfies the demand for a large training dataset for the deep learning method.

Since our model is “train once, apply everywhere”, we test it on ECG recordings from 6 test databases that are not included in the training and validation dataset. In our experiments, we stress the time separation between enrollment and classification because it is an important factor in a practical application that many researchers overlooked. We improve the identification accuracy over long time separation when compared to one published result. We also present the performance of the model against different time separations to compare with future research.

When compared to other state-of-the-art methods, our design slightly underperforms some of the specialized designs under their most favorable test conditions. However, our design is the best among the generalized methods.

## Figures and Tables

**Table 1:** A summary of the related terminologies.

Term	Alternative Term	Description
Enrollment	-	Registering a new ECG into the system
Classification	-	Referring to both verification and identification
Query	-	ECG used for classification
Classification scope	Closed set [18] Gallery [17] Gallery set [15,16]	Collection of enrolled ECGs to be considered during a classification
Individual verification	-	Classifying whether the query matches 1 claimed identity
Scope verification	Identity verification [18] Set verification [15,16]	Classifying whether the query matches identities in the classification scope
Closed identification	-	Identification with the assumption that the query must match 1 identity within the classification scope
Opened identification	-	Closed identification + scope verification

**Table 2:** A summary of the evaluation metrics.

Metric	Alternative Term	Formula/Description
True positive rate (TPR)	True acceptance rate [2] Genuine acceptance rate [19] Recall [4,17] Sensitivity [20]	$\mathrm{TPR}=\frac{TP}{TP+FN}$ [2,4]
False positive rate (FPR)	False acceptance rate [2,14,17,19]	$\mathrm{FPR}=\frac{FP}{FP+TN}$ [2,4]
True negative rate (TNR)	Specificity [17,20]	$\mathrm{TNR}=\frac{TN}{TN+FP}$ [20]
False negative rate (FNR)	False rejection rate [4]	$\mathrm{FNR}=\frac{FN}{FN+TP}$ [4]
Equal error rate (EER) [14,19,20]	-	Error rate when FPR=FNR [14]
Receiver operating characteristics (ROC)	-	Graph of TPR against FPR [19]
Identification accuracy [5,20]	Identification rate [3,17] Recognition accuracy [6]	Rate of correct identification [5]

**Table 3:** A summary of the related works.

Author	Segmentation	Feature	Classification	Data Source	Scope Size	Classification Type	Enrollment to Classification Time	Enrollment Length	Classification Length
[1]	No need	Fiducial	SIMCA	Private	20	Identification	Not specified	Not specified	Not specified
[11]	No need	DWT	Correlation coefficient	Public	18–48	Identification	Not specified	Not specified	Not specified
[8]	Heartbeat	Fiducial	Similarity thresholding	Public	73	Verification	Not specified	30 s	4 s
[14]	R-R and R-peak with fixed length	Fiducial and DWT	Euclidean distance and DTW	Public	20–1119	Verification	Not specified	10 segments	Vary depends on database
[19]	Heartbeat	Fiducial	PCA and Euclidean distance	Public	100	Verification	Not specified	30 s	30 s
[5]	R-peak with fixed length	Fiducial and DWT	Random forest and wavelet distance	Public	18–89	Identification	Not specified	67% of extracted heartbeats	1 heartbeat
[21]	R-peak with fixed length	Learned	CNN	Public	18–23	Identification	Not specified	100 heartbeats	3 heartbeats
[6]	Blind with fixed length	Multi-domain, MFCC, FFT, DCT, Teager, etc.	Channel attention module (CNN)	Public	50–89	Identification	Avg. 63 days for PTBDB, avg. 9 days for EDGIDDB	40 s	4 s
[2]	Heartbeat	DWT	NEWFM	Public	73	Verification	Not specified	15 heartbeats	1 heartbeat
[3]	Blind with fixed length	Learned	CNN	Private	1019	Identification	maximum 6 months	Not specified	5 s
[20]	R-peak with fixed length	Learned	Neural network	Public	90	Verification	9 days for EDGIDDB	Not specified	Not specified
[4]	Blind with fixed length	Fiducial	Random forest	Public	1985	Verification	Not specified	1 m	3 s
[22]	R-peak with fixed length	Learned	RNN	Public	47–89	Identification	Not specified	18 heartbeats	18 heartbeats
[23]	Blind with fixed length	DWT	CNN	Public	18–47	Identification	Not specified	250 × 2-s segments	1 × 2-s segment
[18]	R-peak with fixed length	Learned	CNN	Public	52	Identification	Not specified	10 s	10 s

**Table 4:** ECG databases used to generate training and validation datasets.

Database	Name	Health Condition	Length	Training-Validation Split Ratio
APNEA-ECG	Apnea-ECG Database	Apnea	7–10 h	38:32
LTAFDB	Long Term AF Database	Paroxysmal or sustained atrial fibrillation	24–25 h	48:32
MITDB	MIT-BIH Arrhythmia Database	Arrhythmia	0.5 h	31:16
LTDB	MIT-BIH Long-Term ECG Database	Unspecified	14–22 h	6:1
VFDB	MIT-BIH Malignant Ventricular Ectopy Database	Ventricular tachycardia, ventricular flutter, and ventricular fibrillation	0.5 h	14:8
SLPDB	MIT-BIH Polysomnographic Database	Apnea	80 h	8:8
SVDB	MIT-BIH Supraventricular Arrhythmia Database	Supraventricular arrhythmia	0.5 h	46:32
INCARTDB	St Petersburg INCART 12-lead Arrhythmia Database	Various diagnosis	0.5 h	43:32
FANTASIA	Fantasia Database	Healthy	2 h	24:16
PTB-XL	PTB-XL, a large publicly available electrocardiography dataset	Mix of healthy and various heart conditions	10 s	18,853:32

**Table 5:** ECG databases used for testing.

Database	Name	Total People	Description
AFDB	MIT-BIH Atrial Fibrillation Database [39]	23	Each person has 1 recording. Each recording is at least 8 h long.
NSRDB	MIT-BIH Normal Sinus Rhythm Database [35]	18	Each person has 1 recording. Each recording is at least 8 h long.
STDB	MIT-BIH ST Change Database [40]	28	Each person has 1 recording. Each recording is at least 12 min long.
CEBSDB	Combined measurement of ECG, Breathing, and Seismocardiography [41]	20	Each person has 3 recordings measured in different positions which sum up to at least 55 min long.
PTBDB	PTB Diagnostic ECG Database	290	112 people have 2 or more recordings with valid time label
ECGIDDB	ECG-ID Database	90	89 people have 2 or more recordings with valid time label

**Table 6:** Variables used for short time separation test.

Database	r (s)	v	t (s)	p (s)	n	h
APNEA-ECG	32	12	0	256	64	23
LTAFDB	32	12	0	256	64	20
MITDB	32	12	0	256	64	18
LTDB	32	12	0	256	64	28
VFDB	20	8	-	20	4	89
SLPDB	32	12	-	32	8	112

**Table 7:** Variables used for long time separation test.

Database	r (s)	v	p (s)	n	h	r (s)
ECGIDDB	20	8	20	4	89	20
PTBDB	32	12	32–56	8	112	32

**Table 8:** Variables used for all time separation test.

Database	r (s)	v	p (s)	n	h
AFDB	32	12	256	8	23
CEBSDB	32	12	32	4	20
NSRDB	32	12	256	8	18
STDB	32	12	32	4	28

**Table 9:** Metrics used to evaluate the model performance.

Performance	Metrics
Individual verification	TPR when FPR is at 1%, 5%, and 10%
	EER
	Area under ROC curve
Scope verification	TPR when FPR is at 10%, 20%, and 30%
	EER
	Area under ROC curve
Closed identification	Accuracy

**Table 10:** Performance over short time separation. h: total number of people, TPR: true positive rate, FPR: false positive rate, EER: equal error rate, ID: identification, ROC: receiver operating characteristics.

Database	h	Individual Verification					Scope Verification					ID Accuracy (%)
		TPR (%) When FPR Is at			EER (%)	Area under ROC	TPR (%) When FPR Is at			EER (%)	Area under ROC	
		1%	5%	10%			10%	20%	30%			
AFDB	23	91.17	97.35	98.70	3.44	0.9926	87.09	91.85	94.70	12.06	0.9454	96.20
CEBSDB	20	100.00	100.00	100.00	0.29	0.9989	100.00	100.00	100.00	6.41	0.9794	100.00
NSRDB	18	99.74	100.00	100.00	0.87	0.9979	97.83	99.74	100.00	6.38	0.9654	99.91
STDB	28	92.80	98.60	99.27	3.00	0.9956	90.90	92.97	95.20	9.32	0.9640	96.09
ECGIDDB	89	97.75	98.88	99.72	1.97	0.9966	83.15	95.22	97.19	15.03	0.9226	96.35
PTBDB	112	98.33	99.42	99.78	1.56	0.9984	95.31	98.21	98.77	8.54	0.9689	98.10

**Table 11:** Performance over long time separation.

Database	h	Individual Verification					Scope Verification					ID Accuracy (%)
		TPR (%) When FPR Is at			EER (%)	Area Uder ROC	TPR (%) When FPR Is at			EER (%)	Area Under ROC	
		1%	5%	10%			10%	20%	30%			
ECGIDDB	89	94.94	98.60	98.88	1.97	0.9885	64.89	89.89	93.54	17.56	0.9009	92.70
PTBDB	112	69.47	81.86	90.27	10.19	0.9460	49.78	63.94	74.34	27.43	0.7852	64.16

**Table 12:** Performance comparison of individual verification over short time separation.

Database	Methods	h	FPR (%)	FNR (%)	EER (%)
CEBSDB	Ingale et al. [14]	20	0.00	0.00	0.00
	Ours	20	0.92	0.00	0.29
ECGIDDB	Ingale et al. [14]	89	1.86	0.00	2.00
	Salloum et al. [22]	18	-	-	0.00
	Ours	89	2.50	1.69	1.97
PTBDB	Ingale et al. [14]	290	0.59	0.00	0.50
	Pal et al. [19]	100	1.63	10.00	2.88
	Ours	112	0.85	1.79	1.56

**Table 13:** Performance comparison of individual verification over short time separation.

Database	Methods	h	FPR (%)
CEBSDB	Zhang et al. [23]	23	93.90
	Li et al. [21]	23	90.90
	Ours	23	96.20
ECGIDDB	Zhang et al. [23]	20	99.00
	Li et al. [21]	20	95.00
	Ours	20	100.00
NSRDB	Tan et al. [5]	18	99.98
	Zhang et al. [23]	18	95.10
	Li et al. [21]	18	96.10
	Ours	18	99.91
STDB	Zhang et al. [23]	28	90.30
	Li et al. [21]	28	95.20
	Ours	28	96.09
ECGIDDB	Sellami et al. [11]	40	92.50
	Salloum et al. [22]	89	97.00
	Tan et al. [5]	89	98.79
	Ours	89	96.35
PTBDB	Labati et al. [18]	52	100.00
	Ours	112	98.10

**Table 14:** Performance comparison of individual verification over long time separation.

Database	Methods	h	ID Accuracy (%)
ECGIDDB	Sun et al. [6]	89	85.94
	Ours	89	92.70
PTBDB	Sun et al. [6]	50+	56.93
	Ours	112	64.16

**Figure A1:**
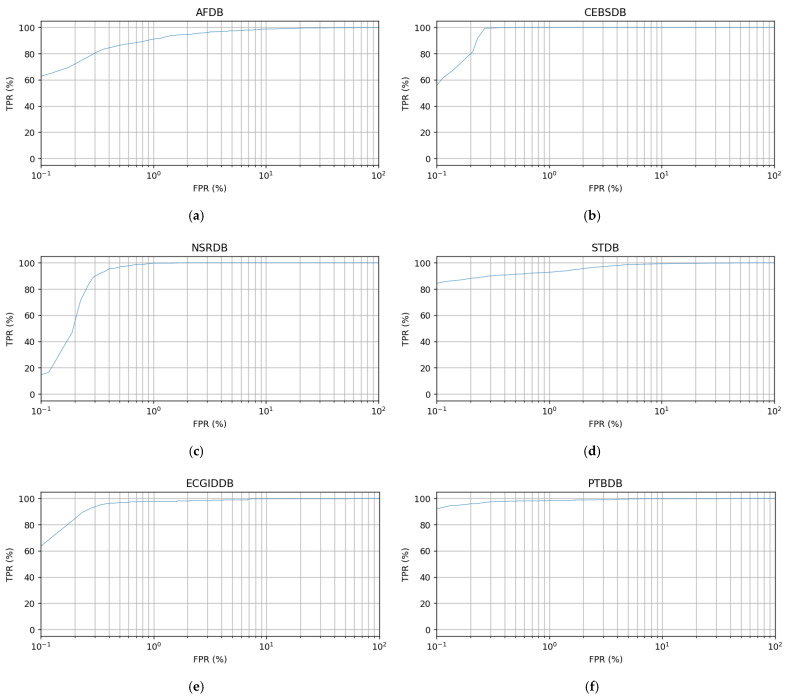
ROC curves are used to evaluate the individual verification performance tested over short time separation for: (**a**) AFDB, (**b**) CEBSDB, (**c**) NSRDB, (**d**) STDB, (**e**) ECGIDDB, (**f**) PTBDB.

**Figure A2:**
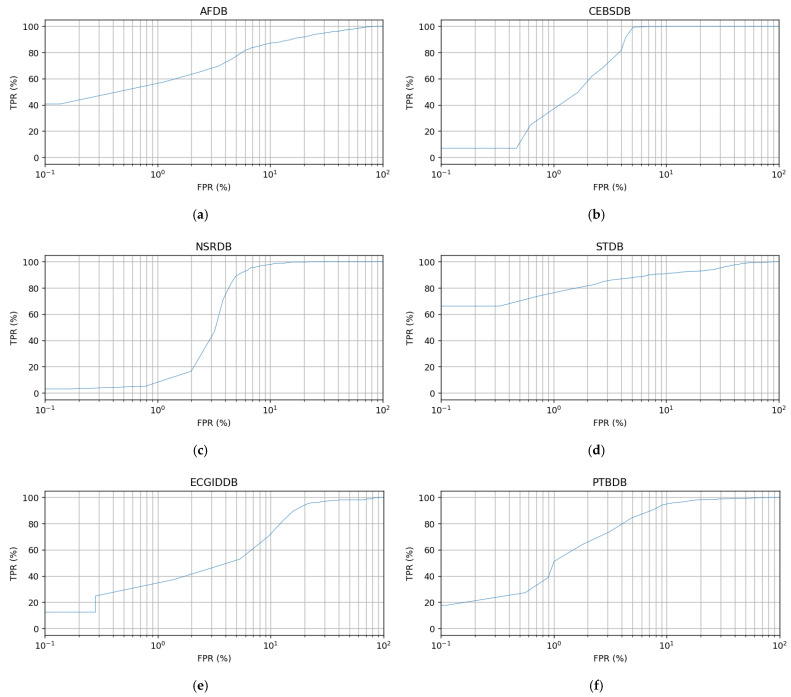
ROC curves are used to evaluate the scope verification performance tested over short time separation for: (**a**) AFDB, (**b**) CEBSDB, (**c**) NSRDB, (**d**) STDB, (**e**) ECGIDDB, (**f**) PTBDB.

**Figure A3:**
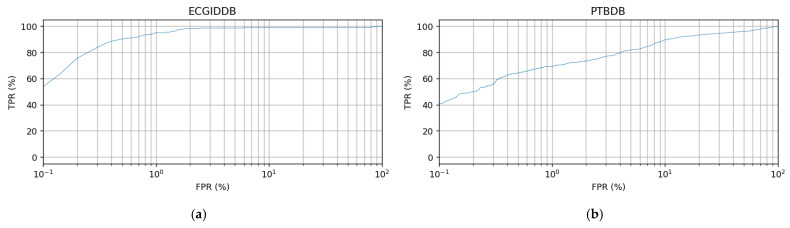
ROC curves are used to evaluate the individual verification performance tested over long time separation for: (**a**) ECGIDDB, (**b**) PTBDB.

**Figure A4:**
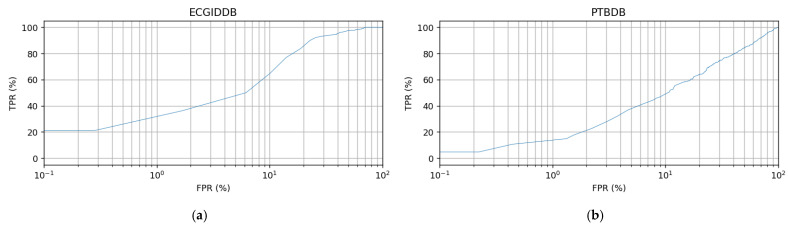
ROC curves are used to evaluate the scope verification performance tested over long time separation for: (**a**) ECGIDDB, (**b**) PTBDB.

**Figure A5:**
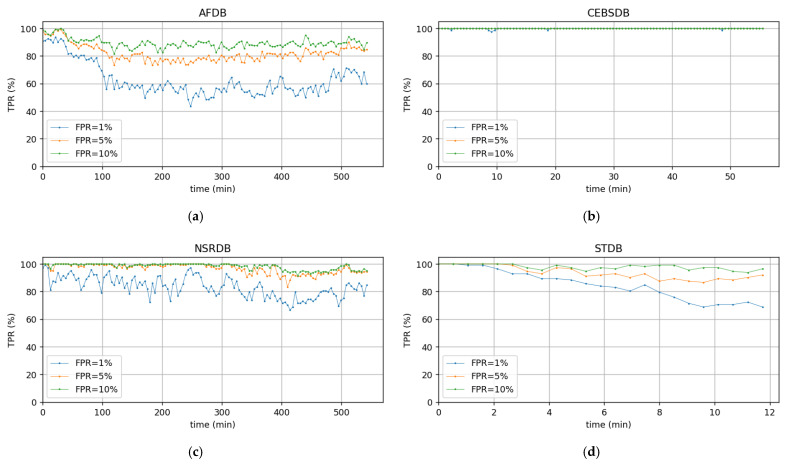
TPR of individual verification is plotted against time separation when FPR is at 1%, 5%, and 10% for: (**a**) AFDB, (**b**) CEBSDB, (**c**) NSRDB, (**d**) STDB.

**Figure A6:**
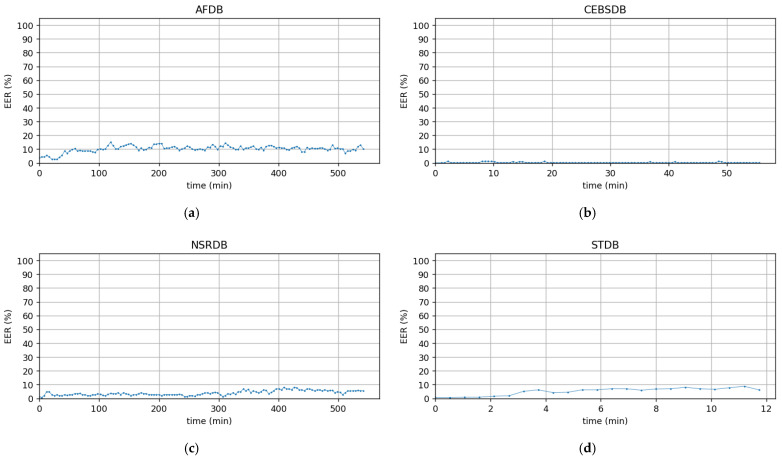
EER of individual verification is plotted against time separation for: (**a**) AFDB, (**b**) CEBSDB, (**c**) NSRDB, (**d**) STDB.

**Figure A7:**
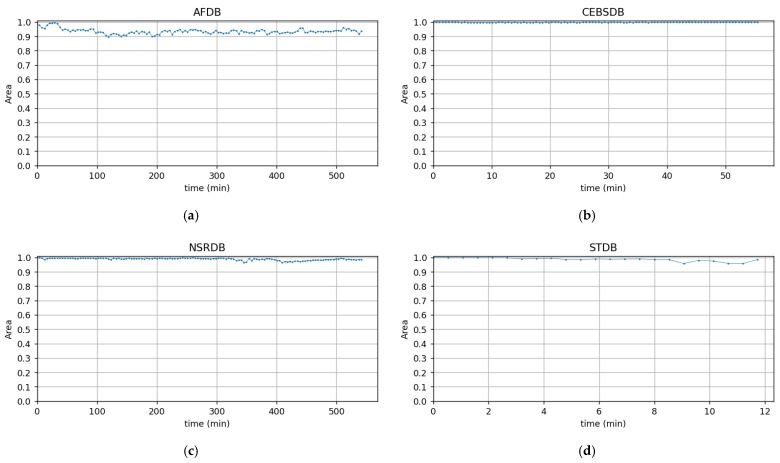
Area under ROC curve of individual verification is plotted against time separation for: (**a**) AFDB, (**b**) CEBSDB, (**c**) NSRDB, (**d**) STDB.

**Figure A8:**
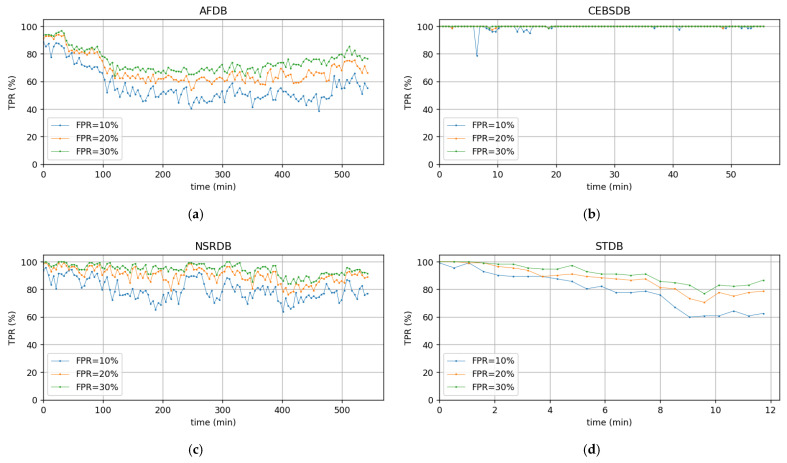
TPR of scope verification is plotted against time separation when FPR is at 10%, 20%, and 30% for: (**a**) AFDB, (**b**) CEBSDB, (**c**) NSRDB, (**d**) STDB.

**Figure A9:**
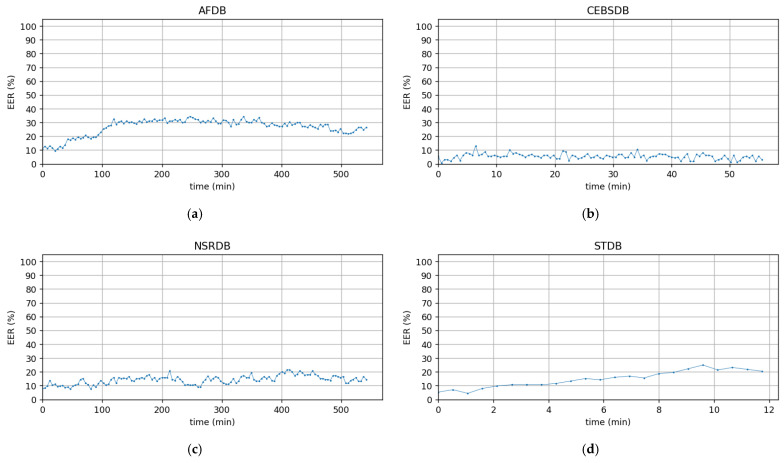
EER of scope verification is plotted against time separation for: (**a**) AFDB, (**b**) CEBSDB, (**c**) NSRDB, (**d**) STDB.

**Figure A10:**
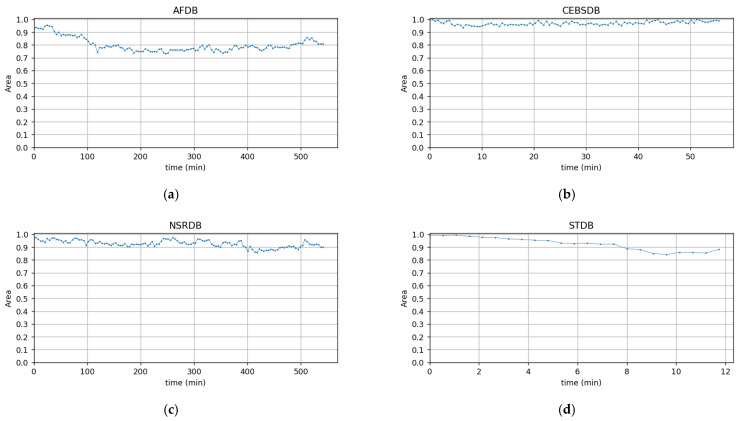
Area under ROC curve of scope verification is plotted against time separation for: (**a**) AFDB, (**b**) CEBSDB, (**c**) NSRDB, (**d**) STDB.

**Figure A11:**
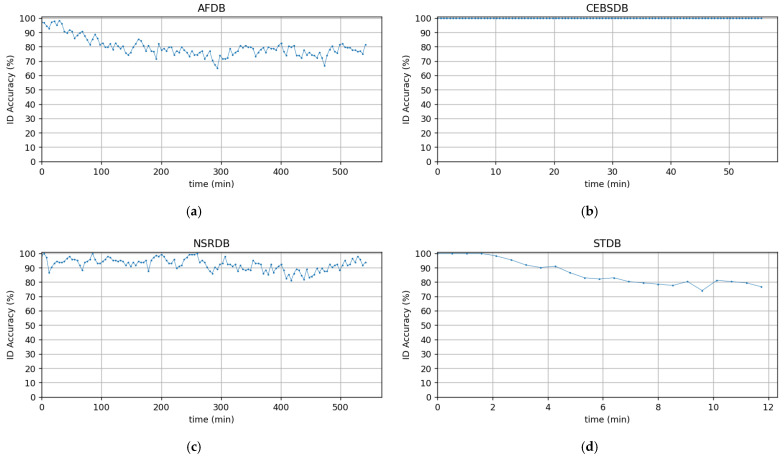
Closed identification accuracy is plotted against time separation for: (**a**) AFDB, (**b**) CEBSDB, (**c**) NSRDB, (**d**) STDB.

## Data Availability

The ECG databases used in this study are publicly available. They can be found here: [https://www.physionet.org/content/apnea-ecg/1.0.0/], [https://www.physionet.org/content/ltafdb/1.0.0/], [https://www.physionet.org/content/mitdb/1.0.0/], [https://www.physionet.org/content/ltdb/1.0.0/], [https://www.physionet.org/content/vfdb/1.0.0/], [https://www.physionet.org/content/slpdb/1.0.0/], [https://www.physionet.org/content/svdb/1.0.0/], [https://www.physionet.org/content/incartdb/1.0.0/], [https://www.physionet.org/content/fantasia/1.0.0/], [https://www.physionet.org/content/ptb-xl/1.0.1/], [https://www.physionet.org/content/afdb/1.0.0/], [https://www.physionet.org/content/nsrdb/1.0.0/], [https://www.physionet.org/content/stdb/1.0.0/], [https://www.physionet.org/content/cebsdb/1.0.0/], [https://www.physionet.org/content/ptbdb/1.0.0/], [https://www.physionet.org/content/ecgiddb/1.0.0/], accessed on 6 February 2022.
